# Effects of Plyometric Jump Training on the Reactive Strength Index in Healthy Individuals Across the Lifespan: A Systematic Review with Meta-analysis

**DOI:** 10.1007/s40279-023-01825-0

**Published:** 2023-03-11

**Authors:** Rodrigo Ramirez-Campillo, Rohit K. Thapa, José Afonso, Alejandro Perez-Castilla, Chris Bishop, Paul J. Byrne, Urs Granacher

**Affiliations:** 1 Exercise and Rehabilitation Sciences Institute, School of Physical Therapy, Faculty of Rehabilitation Sciences, Universidad Andres Bello, 7591538 Santiago, Chile; 2 School of Physical Education and Sports, Rashtriya Raksha University, Gandhinagar, India; 3 Centre for Research, Education, Innovation, and Intervention in Sport (CIFI2D), Faculty of Sport of the University of Porto, Rua Dr. Plácido Costa, 91, 4200-450 Porto, Portugal; 4 Department of Education, Faculty of Education Sciences, University of Almería, 04120 Almería, Spain; 5 London Sport Institute, Middlesex University, The Burroughs, London, UK; 6 Department of Health and Sport Sciences, South East Technological University, Kilkenny Road Campus, Carlow, Ireland; 7 Department of Sport and Sport Science, Exercise and Human Movement Science, University of Freiburg, Sandfangweg 4, 79102 Freiburg i. Br., Germany; 8 SPORT Research Group (CTS-1024), CERNEP Research Center, University of Almería, Almería, Spain

## Abstract

**Background:**

The reactive strength index (RSI) is meaningfully associated with independent markers of athletic (e.g., linear sprint speed) and neuromuscular performance [e.g., stretch–shortening cycle (SSC)]. Plyometric jump training (PJT) is particularly suitable to improve the RSI due to exercises performed in the SSC. However, no literature review has attempted to meta-analyse the large number of studies regarding the potential effects of PJT on the RSI in healthy individuals across the lifespan.

**Objective:**

The aim of this systematic review with meta-analysis was to examine the effects of PJT on the RSI of healthy individuals across the lifespan compared with active/specific-active controls.

**Methods:**

Three electronic databases (PubMed, Scopus, Web of Science) were searched up to May 2022. According to the PICOS approach, the eligibility criteria were: (1) healthy participants, (2) PJT interventions of ≥ 3 weeks, (3) active (e.g., athletes involved in standard training) and specific-active (e.g., individuals using heavy resistance training) control group(s), (4) a measure of jump-based RSI pre-post training, and (5) controlled studies with multi-groups in randomised and non-randomised designs. The Physiotherapy Evidence Database (PEDro) scale was used to assess the risk of bias. The random-effects model was used to compute the meta-analyses, reporting Hedges’ *g* effect sizes (ES) with 95% confidence intervals (95% CIs). Statistical significance was set at *p* ≤ 0.05. Subgroup analyses were performed (chronological age; PJT duration, frequency, number of sessions, total number of jumps; randomization). A meta-regression was conducted to verify if PJT frequency, duration, and total number of sessions predicted the effects of PJT on the RSI. Certainty or confidence in the body of evidence was assessed using Grading of Recommendations Assessment, Development, and Evaluation (GRADE). Potential adverse health effects derived from PJT were researched and reported.

**Results:**

Sixty-one articles were meta-analysed, with a median PEDro score of 6.0, a low risk of bias and good methodological quality, comprising 2576 participants with an age range of 8.1–73.1 years (males, ~ 78%; aged under 18 years, ~ 60%); 42 studies included participants with a sport background (e.g., soccer, runners). The PJT duration ranged from 4 to 96 weeks, with one to three weekly exercise sessions. The RSI testing protocols involved the use of contact mats (*n* = 42) and force platforms (*n* = 19). Most studies reported RSI as mm/ms (*n* = 25 studies) from drop jump analysis (*n* = 47 studies). In general, PJT groups improved RSI compared to controls: ES = 0.54, 95% CI 0.46–0.62, *p* < 0.001. Training-induced RSI changes were greater (*p* = 0.023) for adults [i.e., age ≥ 18 years (group mean)] compared with youth. PJT was more effective with a duration of > 7 weeks versus ≤ 7 weeks, > 14 total PJT sessions versus ≤ 14 sessions, and three weekly sessions versus < three sessions (*p* = 0.027–0.060). Similar RSI improvements were noted after ≤ 1080 versus > 1080 total jumps, and for non-randomised versus randomised studies. Heterogeneity (*I*^2^) was low (0.0–22.2%) in nine analyses and moderate in three analyses (29.1–58.1%). According to the meta-regression, none of the analysed training variables explained the effects of PJT on RSI (*p* = 0.714–0.984, *R*^2^ = 0.0). The certainty of the evidence was moderate for the main analysis, and low-to-moderate across the moderator analyses. Most studies did not report soreness, pain, injury or related adverse effects related to PJT.

**Conclusions:**

The effects of PJT on the RSI were greater compared with active/specific-active controls, including traditional sport-specific training as well as alternative training interventions (e.g., high-load slow-speed resistance training). This conclusion is derived from 61 articles with low risk of bias (good methodological quality), low heterogeneity, and moderate certainty of evidence, comprising 2576 participants. PJT-related improvements on RSI were greater for adults versus youths, after > 7 training weeks versus ≤ 7 weeks, with > 14 total PJT versus ≤ 14 sessions, and with three versus < three weekly sessions.

**Supplementary Information:**

The online version contains supplementary material available at 10.1007/s40279-023-01825-0.

## Key Points

**Table:** 

Plyometric jump training is effective at improving the lower-limb reactive strength index in healthy individuals across the lifespan.
Results of this meta-analysis are based on a total of 2576 participants, from 61 articles with low risk of bias (good methodological quality), low study heterogeneity, and moderate certainty of evidence (GRADE).
Plyometric jump training had a greater impact on the reactive strength index in adults compared with youth.
Plyometric jump training was more effective with > 7 versus ≤ 7 training weeks, > 14 total exercise sessions versus ≤ 14 exercise sessions, and three weekly exercise sessions versus < 3 sessions.

## Introduction

The stretch–shortening cycle (SSC) is a key neuromuscular phenomenon underpinning ballistic jump and plyometric performance [1]. An individual’s ability to utilise the SSC, or the ability of the musculotendinous unit to produce a powerful concentric contraction, immediately following a muscle lengthening action [2, 3], typically occurring in movements where body segments are exposed to impact forces that induce stretch (e.g., drop jump), is termed reactive strength, commonly measured with the reactive strength index (RSI) [2–5]. For jump-related movements, the SSC can be broadly described as fast (e.g., ground contact time (GCT) < 250 ms) or slow (> 250 ms) [2, 3]. For example, a drop jump (also named bounce drop jump) often reports GCT < 250 ms (fast SSC) [4, 6]. The countermovement jump (CMJ) involves a slow SSC movement > 500 ms. Different drop jump types such as the depth jump (also named counter-drop jump) [6] involve GCT > 400 ms [4, 6]. Depending on the type of SSC (fast vs. slow), different physiological responses are expected, involving potentially different long-term exercise-induced adaptations [6, 7]. In addition to the SSC duration, the magnitude of the load that initiates the stretch of the SSC results in the stretch velocity and thus in reflex activity preceding the shortening contraction (i.e., jumping action). The larger the load, for instance through higher drop heights, the faster the stretch velocity and the subsequent reflex activity. Indeed, a slow SSC and low stretch velocity are usually evident during a CMJ, while a fast SSC and a high stretch velocity are typical during the drop jump [3, 4, 6]. Therefore, considering that RSI is meaningfully associated with independent markers of athletic performance (e.g., linear sprint speed) [2], and neuromuscular performance (e.g., SSC) across the life span [1, 7–11], the RSI represents a biological marker of interest during the continuous monitoring process of human athletic performance.

Indeed, the RSI is a metric used to assess an athlete’s ability to produce force rapidly [2], and is traditionally measured during tasks indicative of fast SSC and high stretch velocity, for example, drop jumps aimed at minimising GCT [2, 3]. There is evidence that the RSI can discriminate between slower and faster male field sport athletes, with faster athletes demonstrating up to 48% greater RSI values [12]. In addition, in rugby players, the RSI may discriminate between stronger and weaker athletes, with RSI differences between 0.84 ando 1.39 (effect size values) [13]. In sports with increased jump-related loads (e.g., female volleyball athletes), the RSI also differentiates between athletes of elite versus sub-elite competitive levels [14], and similar findings were recently reported for female gymnasts [15]. Furthermore, to improve change-of-direction performance, training recommendations have been developed using reactive strength as an exercise prescription parameter [16]. A recent meta-analysis [2] noted that the RSI was associated with measures of physical fitness and sports performance. Specifically, the RSI was moderately associated with isometric and dynamic strength (pooled strength measures, *r* = 0.34), endurance performance (*r* = 0.40), acceleration (*r* =  − 0.43), top speed (*r* =  − 0.33), and largely associated with change-of-direction performance (*r* =  − 0.57) [2]. However, the aforementioned meta-analysis [2] reported correlations only and can therefore not infer with regard to cause and effect relations, which is why a meta-analysis is needed that assesses the effects of physical exercise on the RSI.

Plyometric jump training (PJT) is a training method that primarily aims at producing high rates of force development through the SSC, with jump exercises involving shorter (e.g., < 250 ms) or longer (e.g., ≥ 250 ms) ground contact times and maximal jump height/distance (i.e., RSI) as distinctive markers of performance during the training sessions [10, 17]. According to the principle of training specificity, PJT is well suited to improve the RSI through neuromuscular adaptations [18]. Indeed, PJT usually implicates a faster SSC muscle action, allowing a greater concentric work performance than an isolated concentric muscle action, stimulating a higher rate of force development, and force absorption muscle capacities (i.e., eccentric force) [6, 10, 18], an important trait for the improvement of fast SSC actions involving a high stretch velocity (e.g., drop jump).

However, the literature is controversial in as much as some studies reported meaningful PJT effects on the RSI, including studies in youth male non-athletes [19], adult female and male physically active participants [20], and endurance athletes [21, 22], while other studies reported non-significant effects in different populations (e.g., highly trained rugby players) [23–25]. These controversial findings can most likely be explained by methodological differences between studies [26, 27]. For example, although PJT studies usually include jump exercises aimed at reducing contact times and maximizing jump height/distance (i.e., RSI), several studies included jump exercises performed in the slow SSC (e.g., jump box), purposefully manipulated according to the participant’s needs (e.g., reduced impact forces) [10, 28–31]. Other methodological issues related to study heterogeneity comprise subject test/training familiarisation versus no familiarisation, the investigation of study populations with different PJT experience, different programming parameters (e.g., frequency, intensity, time), in addition to different testing procedures and measurement equipment [10, 17]. To account for these methodological limitations and to assess the degree of study heterogeneity, the performance of a systematic review with meta-analysis is timely and has the potential to provide meaningful insights.

A systematic review with meta-analysis provides evidence-based knowledge on the effects of PJT on the RSI [32]. Additionally, such research work helps to detect gaps and limitations in the PJT literature, providing valuable information for scientists and practitioners to follow future research avenues. Indeed, previous research work has been performed to solve controversial findings by systematically aggregating the literature related to PJT. The available meta-analyses focused on the effects of PJT on vertical jump height (e.g., drop jump height) without assessing the specific effects of PJT on the RSI [33, 34]. Similarly, previous reviews analysed training-induced effects on the RSI, although: (1) there was a focus on a myriad of strength and conditioning methods without examining single-mode PJT effects, (2) these studies examined specific populations (e.g., endurance runners; post-rehabilitation athletes; males), and (3) some studies were biased in their systematic review and/or meta-analytical approach (e.g., single-control group sample size not proportionately divided in studies including multiple-intervention groups) [2, 21, 35–41]. Additionally, the potential role of moderators such as participants’ sex, age, and sport, have not been addressed in a meta-analytical approach.

Thus, the primary aim of this systematic review with meta-analysis was to examine the effects of PJT on the RSI of healthy individuals across the lifespan compared with active/specific-active controls.

## Methods

### Procedures

A systematic review with meta-analysis was conducted following the guidelines of the Preferred Reporting Items for Systematic Reviews and Meta-Analyses (PRISMA) [42], and adapted a posteriori to new reporting guidelines (e.g., PRISMA 2020) [43–47] as such changes are expected as the field evolves (e.g., new databases; new concepts/terms). The most relevant adaptations are described in the Electronic Supplementary Material (ESM) (Table S1).

### Literature Search: Administration and Update

We considered recommendations from the two most comprehensive scoping reviews that previously examined the PJT literature [10, 17]. Computerized literature searches were conducted in the electronic databases PubMed, Web of Science, and SCOPUS. The search strategy was conducted using the Boolean operators AND/OR in different combinations with the following keywords (all database fields): “ballistic”, “complex”, “cycle”, “explosive”, “force”, “plyometric”, “shortening”, “stretch”, “training”, and “velocity”. Examples of combinations included: “ballistic” AND “training”; (“ballistic” OR “plyometric” OR “explosive”) AND “training”. Additionally, using the title database field, the following keywords were employed in the search: “jump”, “power” and “training”. After an initial search in April 2017, an account was created by one of the authors (RRC) in each of the respective databases, through which the author received automatically generated email updates regarding the search terms used. The search was refined in May 2019 and August 2021, with updates received daily (if available). Studies were eligible for inclusion, from inception in each database, up to May 2022. The main advantage of this search approach is that it assumes that new knowledge will appear and allow improvements in sport/clinical decision-making. Indeed, the publication rate of PJT studies increased exponentially since 2010 [10, 17]. The same author (RRC) conducted the initial search and removed duplicates. Thereafter, the search results were analysed according to the eligibility criteria (Table 1). The search strategy (code line) for each database and background of search history is described in the ESM (Table S1).

**Table 1:** Selection criteria used in the meta-analysis

Category	Inclusion criteria	Exclusion criteria
Population	Participants with no restrictions on their fitness or competitive level, sex, or age	Participants with health problems (e.g., injuries, recent surgery), precluding participation in a plyometric-jump training programme
Intervention	A plyometric-jump intervention (≥ 3 weeks) training programme, which included unilateral and/or bilateral jumps, which commonly utilizes a pre-stretch or countermovement stressing the stretch–shortening cycle	Exercise interventions not involving plyometric-jump training (e.g., upper-body plyometrics only training interventions) or exercise interventions involving plyometric jump training programmes representing less than 50% of the total training load when delivered in conjunction with other training interventions (e.g., high-load resistance training)
Comparator	Active control group (e.g., athletes participating in standard training schedules). Studies comparing different plyometric jump training approaches (e.g., different intensity) without active or traditional control group will also be considered, as well as specific-active control groups (e.g., involving alternative training methods such as high-load resistance training)	Absence of control group
Outcome	Different specific tests (e.g., drop jump; repeated hops) can be used to assess reactive strength index, and these will be included, as long as a measure for jump-based reactive strength index before and after the training intervention is provided	Lack of baseline and/or follow-up data for reactive strength index. Measures of reactive strength index obtained in actions other than jump-based (e.g., running)
Study design	Multi-arm trials	Single-arm trials/observational studies

In selecting studies for inclusion, a review of all relevant titles was conducted before examination of the abstracts and full texts. Two authors (RRC and RKT) independently screened the titles, abstracts and full texts of the retrieved studies. During the search and review process, potential discrepancies between the two authors regarding inclusion and exclusion criteria (e.g., type of control group, intervention adequacy) were resolved through consensus with a third author (APC).

### Inclusion and Exclusion Criteria

A PICOS (participants, intervention, comparators, outcomes, and study design) approach was used to rate studies for eligibility [42]. Table 1 indicates our inclusion/exclusion criteria. Of note, an evidence-based [10, 48] decision was considered to determine the minimal effective PJT duration (weeks) for the improvement of RSI (i.e., ≥ 3 weeks).

Additionally, only original studies in peer-reviewed and full-text format were eligible to be included in this meta-analysis. Additional exclusion criteria are provided in the ESM (Table S2). Because of expected difficulties with the translation of research articles written in different languages and the fact that 99.6% of the jump training literature is published in English [17], only articles written in English, Spanish, German and Portuguese (i.e., authors’ native languages) were considered for this meta-analysis.

### Data Extraction

When extracting RSI data from the included studies, we considered previous recommendations [2, 41]. Therefore, the effects of PJT compared to active (e.g., athletes participating in standard training schedules, participants involved in regular physical education courses or classes) and/or specific-active (e.g., involving alternative training methods such as high-load resistance training) controls on the RSI and its constituent parts (e.g., jump height, GCT) were assessed. Measures of the RSI include (but are not limited to) different specific tests (e.g., drop jumps, repeated hops, CMJ), indices (e.g., mm/ms, cm/ms) or calculation procedures (e.g., jump height, flight time, contact time, time to take-off (e.g., modified RSI obtained from CMJ movements using a force platform)). The RSI has shown moderate to strong levels of reliability (intra-class correlation coefficient = 0.57–0.99; coefficient of variation = 3.0–14%) across a range of populations [2], which is essential to ensure strong consistency between the analysed studies within a meta-analysis [42].

Pre- and post-intervention, means and standard deviation of the dependent variables were extracted from the included studies using Microsoft Excel (Microsoft Corporation, Redmond, WA, USA). For studies reporting values other than means and standard deviation (e.g., median, range, interquartile range, standard error values), conversion was applied as previously recommended [49–51]. Appropriate statistical software was used for different data formats (Comprehensive Meta-Analysis Software, Version 2, Biostat, Englewood, NJ, USA). When the required data were not clearly or completely reported, the authors of the respective studies were contacted for clarification purposes. If no response was obtained from the authors (after two attempts, with a between-attempts waiting time of 72 h) or the authors did not provide the requested data, the study outcome was excluded from further analysis. When data were displayed in a figure and no numerical data were provided by the authors, validated (*r* = 0.99, *p* < 0.001) [52] software (WebPlotDigitizer, version 4.5; https://apps.automeris.io/wpd/) was used to derive numerical data from the respective figures. One author (RRC) performed data extraction and a second author (RKT) provided confirmation, and any discrepancies between them (e.g., mean value for a given outcome) were resolved through consensus with a third author (PB).

### Risk of Bias of the Included Studies

The Physiotherapy Evidence Database (PEDro) scale was used to assess the risk of bias in the included studies, which were rated from 0 (lowest quality) to 10 (highest quality). The validity and reliability of the PEDro scale have been established previously [53–55]. Moreover, the PEDro scale is the most frequently used metric in the PJT literature [10, 56, 57]. Despite being termed a “methodological quality” scale, its items mostly assess factors related to the risk of bias of studies. Accordingly, it helps to make comparisons between meta-analyses. Considering that it is not possible to satisfy all scale items in PJT interventions [58] and as outlined in previous systematic reviews in the sub-field of PJT, the overall risk of bias of PJT studies was interpreted using the following convention [56, 58–60]: ≤ 3 points was considered as “poor” quality (i.e., high risk of bias), 4–5 points was considered as “moderate” quality, while 6–7 points and 8–10 points were considered as “good” and “excellent” quality, respectively. For practical purposes and given the nature of the research field, we considered studies with ≥ 6 points to have low risk of bias [61]. If trials were already rated and listed in the PEDro database, the respective scores were adopted. Two authors (RRC and RKT) assessed the risk of bias for each included study independently, and any discrepancies between them were resolved via consensus with a third author (UG). To reduce high risk of bias in the analysis, a posteriori, a decision was made regarding the exclusion of studies rated with ≤ 3 points.

### Summary Measures, Synthesis of Results, and Publication Bias

According to the Cochrane Handbook [62], meta-analyses can be computed with as few as two studies [63]; we performed our analyses if ≥ three studies were available [9, 64, 65]. Means and standard deviations from pre and post values were taken to compute effect sizes (ES; i.e., Hedges’ *g*) for RSI in the PJT and active/specific-active control groups. Data were standardised using post-intervention standard deviation values. The DerSimonian and Laird random-effects model was used to account for differences between studies that might affect the PJT effects [66, 67]. The ES values are presented with 95% confidence intervals (95% CIs). Calculated ES were interpreted using the following scale: < 0.2 trivial, 0.2–0.6 small, > 0.6–1.2 moderate, > 1.2–2.0 large, > 2.0–4.0 very large, > 4.0 extremely large [68]. In studies including more than one intervention group, the sample size in the control group was proportionately divided to facilitate comparisons across multiple groups [69]. The impact of study heterogeneity was assessed using the *I*^2^ statistics, with values of < 25%, 25–75%, and > 75% representing low, moderate, and high levels of heterogeneity, respectively [70]. The risk of publication bias was explored for continuous variables (≥ 10 studies per outcome) [71–73] using the extended Egger’s test [73]. To adjust for risk of publication bias, a sensitivity analysis was conducted using the trim and fill method [74], with L0 as the default estimator for the number of missing studies [75]. All analyses were carried out using the Comprehensive Meta-Analysis Software (Version 2, Biostat, Englewood, NJ, USA). Statistical significance was set at *p* ≤ 0.05.

### Additional Analyses

#### Subgroup Analyses

Potential sources of heterogeneity likely to influence the effects of training were selected a priori. However, the exact number of subgroups became evident only after the identification of all studies eligible for inclusion. As adaptive responses to PJT programs may be affected by the individual’s age [76–78], this factor was considered as a potential moderator variable. Accordingly, the results derived from studies conducted in groups of adult participants (i.e., groups with a mean age ≥ 18 years) were compared to the results derived from studies conducted in groups of youth participants (i.e., groups with a mean age < 18 years).

#### Single Training Factor Analyses

Potential sources of study heterogeneity arising from PJT configurations were selected a priori. Single training factor analyses were computed for the program duration (intervention duration and total number of training sessions) [33] and training frequency (number of weekly exercise sessions) [79], based on the reported impact of these variables on adaptations following PJT. Additional moderators such as total number of jumps were also considered if the studies provided such data.

When appropriate, subgroup analyses and single training factor analyses were analysed using the median split technique [80–82]. The median was calculated if at least three studies provided data for a given moderator. Of note, when two experimental groups (with the same information for a given moderator) were included in a study, only one of the groups was considered to avoid an augmented influence of the study on the median calculation. In addition, instead of using a global median value for a given moderator (e.g., median age, derived from all included studies), median values were calculated considering only those studies that provided data for the analysed outcome. When the median split technique was found not to be appropriate, a logically defensible rationale was used for subgroup analysis.

#### Randomised versus Non-randomised Trials

We conducted a subgroup analysis contrasting randomised versus non-randomised studies.

#### Sensibility Analyses

We performed sensitivity analyses to assess the robustness of the summary estimates (e.g., *p* value, ES, *I*^2^). To examine the effects of each result from each study on the overall findings, results were analysed with each study deleted from the model (automated leave-one-out analysis).

#### Meta-Regression

A multivariate DerSimonian and Laird random-effects model meta-regression was conducted to verify if any of the training variables (frequency, duration and total number of sessions) explained the effects of PJT on the RSI. The computation of meta-regression was performed with at least ten studies per covariate [71].

#### Certainty of Evidence

Two authors (JA and RRC) rated the certainty of evidence (i.e., high, moderate, low, very low) using the Grading of Recommendations, Assessment, Development and Evaluation (GRADE) [83–85]. The evidence started at a high level of certainty (per outcome), but was downgraded based on the following criteria: (1) risk of bias in studies: judgments were downgraded by one level if the median PEDro scores were moderate (< 6) or by two levels if they were poor (< 4); (2) indirectness: low risk of indirectness was attributed by default due to the specificity of populations, interventions, comparators and outcomes being guaranteed by the eligibility criteria; (3) risk of publication bias: downgraded by one level if there was suspected publication bias; (4) inconsistency: judgements were downgraded by one level when the impact of statistical heterogeneity (I^2^) was high (> 75%); (5) imprecision: one level of downgrading occurred whenever < 800 participants were available for a comparison [86] and/or if there was no clear direction of the effects. When both were observed, certainty was downgraded by two levels.

#### Adverse Effects

In addition, considering the potential adverse health effects derived from the inadequate implementation of PJT interventions, a qualitative analysis of such potential effects was included.

### Registration

The protocol for this systematic review with meta-analysis was published in the Open Science Framework (OSF) on 16 May 2022 (Project: https://osf.io/t9pjg/; Registration: https://osf.io/8fw3q).

## Results

### Study Selection

**Fig. 1:**
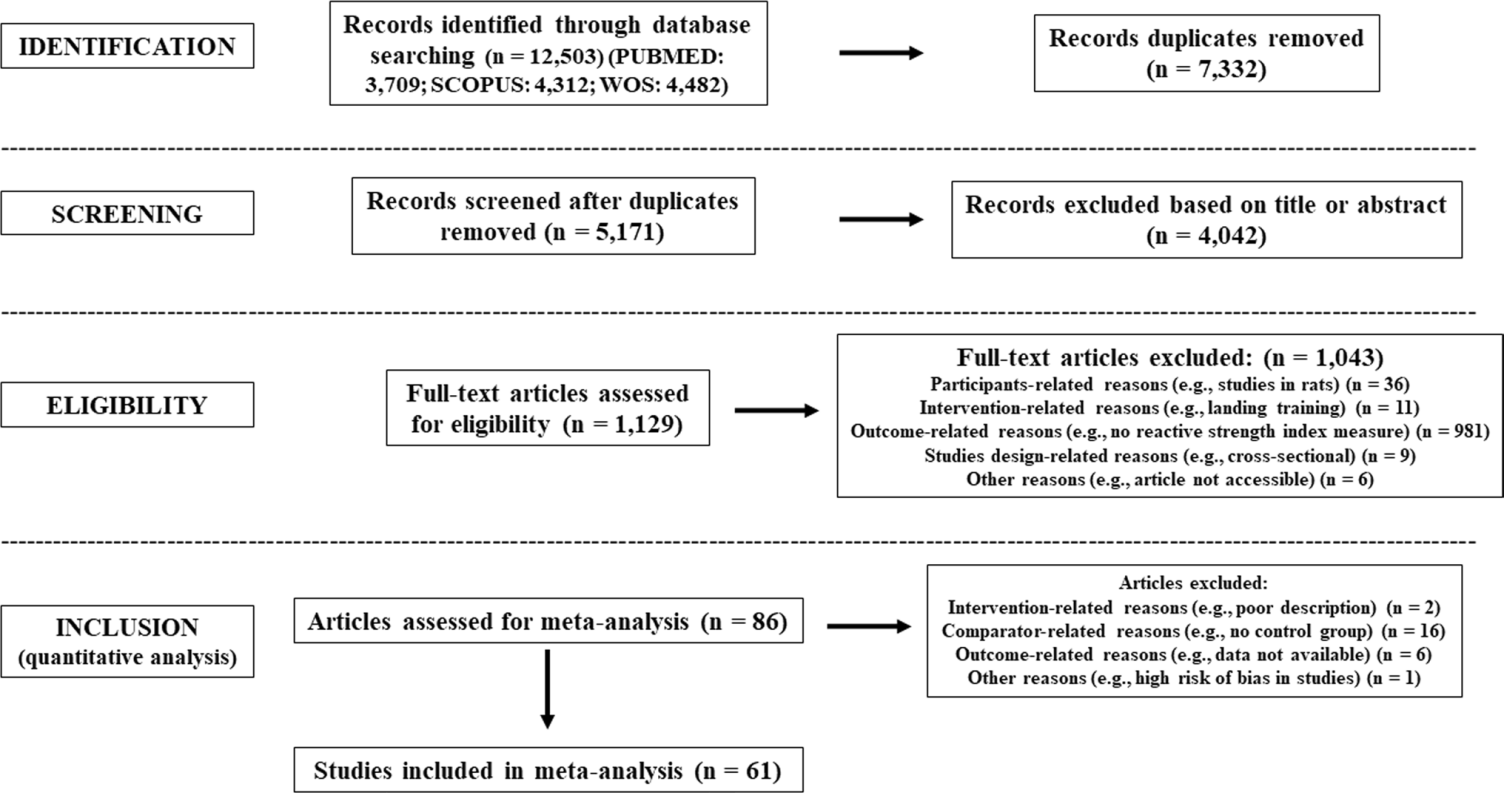
Flow diagram of the systematic search process

The search process in the databases identified 12,503 studies. Figure 1 provides a flow chart illustrating the study selection process.

Duplicate studies were removed (*n* = 7332). After study titles and abstracts were screened, 4042 studies were removed and 1129 full texts were screened. From the 86 studies assessed to be eligible for inclusion, 25 full texts were excluded (see Fig. 1 for exclusion reasons). Finally, 61 studies were considered eligible for this meta-analysis [19, 25, 87–145], of which 60 were written in English, and one in German [144].

### Risk of Bias of the Included Studies

According to the PEDro checklist results (Table 2), the median (i.e., non-parametric) score was 6.0 (low risk of bias—good quality), with 27 studies attaining 4–5 points (some risk of bias—moderate quality), 32 studies attaining 6–7 points, and two studies with 8–10 points (low risk of bias; good and excellent quality, respectively). The two independent reviewers who performed the methodological appraisal of the included studies achieved a Spearman correlation (i.e., non-parametric data) agreement of 0.91.

**Table 2:** Rating of studies according to the Physiotherapy Evidence Database (PEDro) scale

	1	2	3	4	5	6	7	8	9	10	11	Score^a^	Study quality
Ando et al. [85]	1	1	0	0	0	0	0	1	1	1	1	5	Moderate
Beattie et al. [86]	1	0	0	1	0	0	0	1	1	1	1	5	Moderate
Bogdanis et al. [24]	1	1	0	1	0	0	0	1	1	1	1	6	Good
Byrne et al. [88]	1	1	0	0	0	0	0	1	1	1	1	5	Moderate
Byrne et al. [87]	1	1	0	1	0	0	0	1	1	1	1	6	Good
Chaabene et al. [89]	1	0	0	1	0	0	0	1	1	1	1	5	Moderate
Chaouachi et al. [90]	1	1	0	1	0	0	0	1	1	1	1	6	Good
Coşkun et al. [91]	1	1	0	0	0	0	0	1	1	1	1	5	Moderate
Dallas et al. [92]	1	1	0	0	0	0	0	1	1	1	1	5	Moderate
Davies et al. [93]	1	0	0	0	0	0	0	1	1	1	1	4	Moderate
Faude et al. [94]	1	1	0	1	0	0	0	0	1	1	1	5	Moderate
Fiorilli et al. [95]	1	1	0	1	0	0	0	1	1	1	1	6	Good
Garcia-Pinillos et al. [96]	1	1	0	1	0	0	0	1	1	1	1	6	Good
Hoffren-Mikkola et al. [97]	1	1	1	1	0	0	0	1	1	1	1	7	Good
Hutchinson et al. [98]	1	0	0	0	0	0	0	1	1	1	1	4	Moderate
Jeffreys et al. [99]	1	1	0	0	0	0	0	1	1	1	1	5	Moderate
Katsikari et al. [100]	1	1	0	0	0	0	0	1	1	1	1	5	Moderate
Keiner et al. [101]	1	0	0	1	0	0	0	1	1	1	1	5	Moderate
Laurent et al. [102]	1	0	0	0	0	0	0	1	1	1	1	4	Moderate
Li et al. [103]	1	1	0	1	0	0	0	1	1	1	1	6	Good
Lloyd et al. [105]	1	0	0	0	0	0	0	1	1	1	1	4	Moderate
Lloyd et al. [104]	1	1	0	0	0	0	0	1	1	1	1	5	Moderate
Lovecchio et al. [106]	1	1	0	1	0	0	0	1	1	1	1	6	Good
Lum et al. [107]	1	1	0	0	0	0	0	1	1	1	1	5	Moderate
Makhlouf et al. [108]	1	1	0	1	0	0	0	1	1	1	1	6	Good
Marina and Jemni [109]	1	0	0	0	0	0	0	1	1	1	1	4	Moderate
Markovic et al. [110]	1	1	0	0	0	0	0	1	1	1	1	5	Moderate
Meylan and Malatesta [111]	1	0	0	1	0	0	0	1	1	1	1	5	Moderate
Newton et al. [113]	1	1	0	1	0	0	0	1	1	1	1	6	Good
Newton et al. [112]	1	0	0	0	0	0	0	1	1	1	1	4	Moderate
Nitzsche et al. [114]	1	0	0	0	0	0	0	1	1	1	1	4	Moderate
Raedegard et al. [115]	1	1	0	1	0	0	0	1	1	1	1	6	Good
Ramirez-Campillo et al. [116]	1	1	1	1	0	0	0	1	1	1	1	7	Good
Ramirez-Campillo et al. [117]	1	1	1	1	0	0	0	1	1	1	1	7	Good
Ramirez-Campillo et al. [118]	1	1	1	1	0	0	0	1	1	1	1	7	Good
Ramirez-Campillo et al. [119]	1	1	1	1	0	0	0	1	1	1	1	7	Good
Ramirez-Campillo et al. [120]	1	1	1	1	0	0	0	1	1	1	1	7	Good
Ramirez-Campillo et al. [121]	1	1	1	1	0	0	0	1	1	1	1	7	Good
Ramirez-Campillo et al. [122]	0	1	1	1	1	1	1	1	0	1	1	9	Excellent
Ramirez-Campillo et al. [123]	1	1	1	1	0	0	0	1	1	1	1	7	Good
Ramirez-Campillo et al. [126]	1	1	0	1	0	0	0	1	1	1	1	6	Good
Ramirez-Campillo et al. [127]	1	1	1	1	0	0	0	1	1	1	1	7	Good
Ramirez-Campillo et al. [124]	1	1	1	1	0	0	0	1	1	1	1	7	Good
Ramirez-Campillo et al. [125]	1	1	1	1	0	0	0	1	1	1	1	7	Good
Ramirez-Campillo et al. [128]	1	1	1	1	0	0	0	1	1	1	1	7	Good
Ramirez-Campillo et al. [130]	1	1	1	1	0	0	0	1	1	1	1	7	Good
Ramirez-Campillo et al. [129]	1	1	1	1	0	0	0	1	1	1	1	7	Good
Ramirez-Campillo et al. [21]	1	1	0	1	0	0	0	1	1	1	1	6	Good
Romero et al. [131]	1	1	0	1	0	0	0	1	1	1	1	6	Good
Rosas et al. [132]	1	1	1	0	1	1	1	1	1	1	1	9	Excellent
Rosas et al. [133]	1	1	0	1	0	0	0	1	1	1	1	6	Good
Salonikidis and Zafeiridis [134]	1	1	0	0	0	0	0	1	1	1	1	5	Moderate
Smilios et al. [135]	1	0	0	0	0	0	0	1	1	1	1	4	Moderate
Sortwell et al. [136]	1	1	0	1	0	0	0	1	1	1	1	6	Good
Sporri et al. [137]	1	1	0	0	0	0	0	1	1	1	1	5	Moderate
Taube et al. [138]	1	1	0	1	0	0	0	1	1	1	1	6	Good
Tottori and Fujita [139]	1	1	0	1	0	0	0	1	1	1	1	6	Good
Uzelac-Sciran et al. [140]	1	1	0	0	0	0	0	1	1	1	1	5	Moderate
Vera-Asaoka et al. [141]	1	1	0	1	0	0	1	1	1	1	1	7	Good
Witassek et al. [142]	1	0	0	1	0	0	0	1	1	1	1	5	Moderate
Young et al. [143]	1	1	0	0	0	0	0	1	1	1	1	5	Moderate

^a^From a possible maximal score of 10. A detailed explanation for each PEDro scale item can be accessed at https://www.pedro.org.au/english/downloads/pedro-scale; In brief: item 1, eligibility criteria were specified; item 2, participants were randomly allocated to groups; item 3, allocation was concealed; item 4, the groups were similar at baseline; item 5, there was blinding of all participants regarding the plyometric jump training programme being applied; item 6, there was blinding of all coaches responsible for the application of plyometric jump training programme regarding its aim toward the improvement of reactive strength index; item 7, there was blinding of all assessors involved in measurement of reactive strength index; item 8, measures of reactive strength index were obtained from more than 85% of participants initially allocated to groups; item 9, all participants for whom reactive strength index was available received the treatment or control condition as allocated or, data for reactive strength index were analysed by “intention to treat”; item 10, the results of between-group statistical comparisons are reported for reactive strength index; and item 11, point measures and measures of variability for reactive strength index are provided

### Study Characteristics

The participant characteristics and the PJT programs of the included studies are detailed in Table 3.

**Table 3:** Descriptive characteristics of participants and plyometric jump training interventions

	Rand	Sex	Age (y)	Body mass (kg)	Height (cm)	Sport	Fit	Fr	Weeks	NTJ
Ando et al. [85]	Yes	M	22.0	66.9/64.8	170.3/176.1	NA	NR	3	8	2400
Beattie et al. [86]	No	M	29.5	72.8	183.0	Runners	Mo-H	2	20	570
Bogdanis et al. [24]	Yes	F	8.1	28.7	129.3	Gym	Mo	2	8	2464
Byrne et al. [87]	Yes	M	21.8	76.6	179.0	Hurling	Mo	2	7	252
Byrne et al. [88]	Yes	M	20.8/23.8^a^	82.0/86.3	178.0/184.0	Mixed	Mo	2	8	660
Chaabene et al. [89]	No	F	15.9	62.8	164.0	Handball	Mo	2	8	1600
Chaouachi et al. [90]	Yes	M	13.3/13.7	45.9	158.0/161.5	NA	N	3	8	1872/2340
Coşkun et al. [91]	Yes	M	20.4	NR	NR	PES	NR	3	8	2448
Dallas et al. [92]	Yes	F	8.4/13.9	28.4/48.9	137.9/159.6	Gym/Tae	Mo	2	4	784
Davies et al. [93]	No	F	11.7/14.3	42.9/59.9	154.0/166.3	NA	N	2	7	324 + 1880 m
Faude et al. [94]	Yes	Mix	22.5	76.8	179.0	Soccer	Mo	2	7	~ 360
Fiorilli et al. [95]	Yes	M	13.4	52.1	168.0	Soccer	Mo	2	6	720
Garcia-Pinillos et al. [96]	Yes	Mix	27.2	66.0	172.0	Runners	N-Mo	3–4	10	8667/12,138
Hoffren-Mikkola et al. [97]	Yes	M	73.1	76.8	170.5	NA	L-Mo	3	11	2970/3960
Hutchinson et al. [98]	NR	F	16.0	NR	NR	Gym	H	2	4	1920 + 96 pool laps
Jeffreys et al. [99]	Yes	M	20.3	91.6	182.0	Rugby	Mo-H	2	6	480/1920
Katsikari et al. [100]	Yes	F	9.0/11.0	38.5	145.0	NA	N	2	10	< 2080
Keiner et al. [101]	No	M	9.5/11.5	35.7/38.7	140.0/146.3	Soccer	Mo	1	96	≥ 2880
Laurent et al. [102]	No	Mix	19.0/26.0	68.7/69.7	180.5/180.9	NA	N-Mo	2	10	2980
Li et al. [103]	NR	M	22.2	63.1	178.2	Runners	Mo-H	3	8	1296
Lloyd et al. [104]	Yes	M	12.7/16.4	53.5/67.8	158.3/179.5	NA	N	2	6	486/958
Lloyd et al. [105]	Yes	M	9.4/15.3	32.6/65.0	133.2/174.4	NA	N	2	4	740
Lovecchio et al. [106]	Yes	M	14.0/15.0	62.6	173.0	NA	N	2	6	4320
Lum et al. [107]	Yes	Mix	37.0	62.7	171.0	Runners	Mo	2	6	1050
Makhlouf et al. [108]	Yes	M	11.1/11.3	36.2/36.9	145.4/147.9	Soccer	Mo	2	8	1826
Marina and Jemni [109]	No	F	11.7	30.2	136.1	Gym	H	1	12 + 12^b^	14,784 + 6960 s
Markovic et al. [110]	Yes	M	20.1	76.7	181.0	NA	Mo-H	3	10	1800
Meylan and Malatesta [111]	No	M	13.3	48.6	159.0	Soccer	N-Mo	2	8	> 768
Newton et al. [112]	No	F	20.0	70.5	180.2	Volleyball	Mo-H	2	4	108
Newton et al. [113]	Yes	M	19.0	84.0	189.0	Volleyball	H	2	8	576
Nitzsche et al. [114]	No	F	12.1/12.6	37.2/37.4	147.6/148.0	Gym	N	3	6	1080
Raedegard et al. [115]	Yes	M	22.6	82.5	182.3	Soccer	Mo	2	6	1032
Ramirez-Campillo et al. [116]	Yes	M	21.8/22.4	76.0/80.1	173.9/176.6	RT	Mo-H	2	8	1080
Ramirez-Campillo et al. [117]	Yes	M	16.9	64.9	172.3	Soccer	Mo	2	7	1944
Ramirez-Campillo et al. [118]	Yes	M	12.1/12.9	44.4/45.6	154.0/159.3	Soccer	Mo	2	8	810
Ramirez-Campillo et al. [120]	Yes	M	10.9/15.6	40.8/63.6	148.0/169.0	Soccer	Mo	2	6	1008
Ramirez-Campillo et al. [119]	Yes	M	13.2	48.6	154.0	Soccer	N-Mo	2	7	840
Ramirez-Campillo et al. [121]	Yes	M	13.1/13.9	46.7/47.2	153.0	Soccer	Mo	2	7	906
Ramirez-Campillo et al. [122]	Yes	F	22.9/23.1	56.8/60.4	162.0/164.0	Soccer	Mo	2	6	1440
Ramirez-Campillo et al. [123]	Yes	F/M	20.4/22.4	60.7/68.4	161.0/171.0	Soccer	Mo	2	6	1440
Ramirez-Campillo et al. [124]	Yes	M	12.8/13.0	53.8/53.9	160.0/161.0	Soccer	Mo	2	6	1440
Ramirez-Campillo et al. [125]	Yes	M	11.2/11.6	40.0/44.6	144.0/150.0	Soccer	Mo	2	6	1440/1610
Ramirez-Campillo et al. [126]	Yes	M	11.0/11.6	43.5/45.0	144.0/147.0	Soccer	Mo	2	6	1080/2160
Ramirez-Campillo et al. [127]	Yes	M	14.1/14.2	50.3/51.8	158.0/159.0	Soccer	N-Mo	2	6	2400
Ramirez-Campillo et al. [128]	Yes	M	10.3/10.4	37.0/38.0	141.0/142.0	Soccer	Mo	2	7	840
Ramirez-Campillo et al. [129]	Yes	M	13.2	47.9	154.0	Soccer	Mo	2	7	840
Ramirez-Campillo et al. [130]	Yes	Mix	22.1	60.0	NR	Runners	H	2	6	720
Ramirez-Campillo et al. [21]	Yes	M	16.9	NR	NR	NA	N	2	7	780/1560
Romero et al. [131]	Yes	F	12.7/16.3	40.9/54.0	145.8/153.9	Mixed	N	2	6	1590
Rosas et al. [132]	Yes	F	22.8/24.3	58.1/61.1	162.0/164.0	Soccer	N	2	6	1440
Rosas et al. [133]	Yes	M	12.1/12.3	45.0/47.3	150.0	Soccer	Mo	2	6	1152
Salonikidis and Zafeiridis [134]	Yes	M	21.1	71.7	174.0	Tennis	N-Mo	3	9	> 5832
Smilios et al. [135]	NR	M	22.7	72.7	176.4	RT	N	2	6	360/660/840
Sortwell et al. [136]	Yes	Mix	7.4	28.8/30.3	126.3/128.1	NA	NR	2	8	1140
Sporri et al. [137]	Yes	M	22.2	77.7	180.0	Mixed	N-Mo	3	8	≥ 1426
Taube et al. [138]	Yes	Mix	24.0/25.0	68.0/69.0	177.0/179.0	Mixed	N	3	4	396
Tottori and Fujita [139]	Yes	M	10.0	32.1	138.6	NA	N	1	8	795
Uzelac-Sciran et al. [140]	Yes	M	13.1/14.0	49.4/68.5	159.8/176.3	NA	N	2	8	1336
Vera-Asaoka et al. [141]	Yes	M	11.2/14.4	36.8/54.7	143.0/163.0	Soccer	Mo	2	7	840
Witassek et al. [142]	No	Mix	22.0/25.0	NR	NR	NA	NR	3	8	NR
Young et al. [143]	Yes	M	19.0/34.0	78.5	178.6	Mixed	NR	3	6	468

*F* female, *Fit* fitness, *Fr* frequency of plyometric-jump training sessions per week, *Gym* gymnasts, *H* high, *L* low, *M* male, *Mo* moderate, *N* normal, *NA* not applicable, *NR* not reported, *NTJ* number of total jumps (unless stated differently), *Rand* randomised, *RT* resistance training, *Taek* taekwondo

^a^Values reported as XX.X/XX.X indicates that a range of values was reported in the respective study or that two or more experimental groups were included

^b^Authors applied 12 weeks of plyometric jump training, followed by a wash-out period, and then another intervention period of 12 weeks

Overall, 61 studies were included. Twenty-two studies examined soccer players, 18 studies non-athletes (including resistance-trained participants and physical education students), five studies endurance runners, six studies mixed sports (e.g., basketball, rugby, hurling, Gaelic football, and soccer), four studies gymnasts, two studies volleyball players, one study handball players, one study hurling athletes, one study tennis, and one study rugby players, with a total of 2576 participants with an age range of 8.1–73.1 years. With regards to the study participants, 1509 individuals participated in the intervention groups (102 groups) and 1067 participated in the control groups (73 groups). Among the 73 control groups, seven groups were specific-active controls, and the other 66 groups were active controls. Sixty-one experimental groups (and their respective controls) involved participants with a mean age of < 18 years (Table 3). Regarding participants' sex, eight studies reported a mixed sample of male and females [*n* = 201 (8% of total participants)], 17 groups involved females only [*n* = 385 (15% of total participants)], and 75 groups involved males [*n* = 1990 (77% of total participants)] (Table 3). Training duration in the intervention and control groups ranged from 4 to 96 weeks (Table 3), although most studies lasted 6 weeks, with a median value of 7 weeks. The frequency of weekly training sessions ranged from one to three sessions per week (Table 3).

The testing protocols involved mostly drop jumps (*n* = 47 studies), vertical hop/rebound jumps (*n* = 12), hurdle jumps (*n* = 1), and CMJs (*n* = 1). Different RSI parameters were found including mm/ms (*n* = 25), m/s (*n* = 10), cm/s (*n* = 8), cm/ms (*n* = 4), ms/ms (*n* = 3), s/s (*n* = 1). In a further ten studies, the authors did not provide specific information and mentioned only that the RSI was calculated from jump height and contact time. Different jump test apparatuses were used including contact mats (*n* = 37), contact mats using an optical (e.g., infrared photoelectric cells) measurement system (*n* = 5), and force platforms (*n* = 19).

### Results of the Meta-analysis

#### Reactive Strength Index

**Fig. 2:**
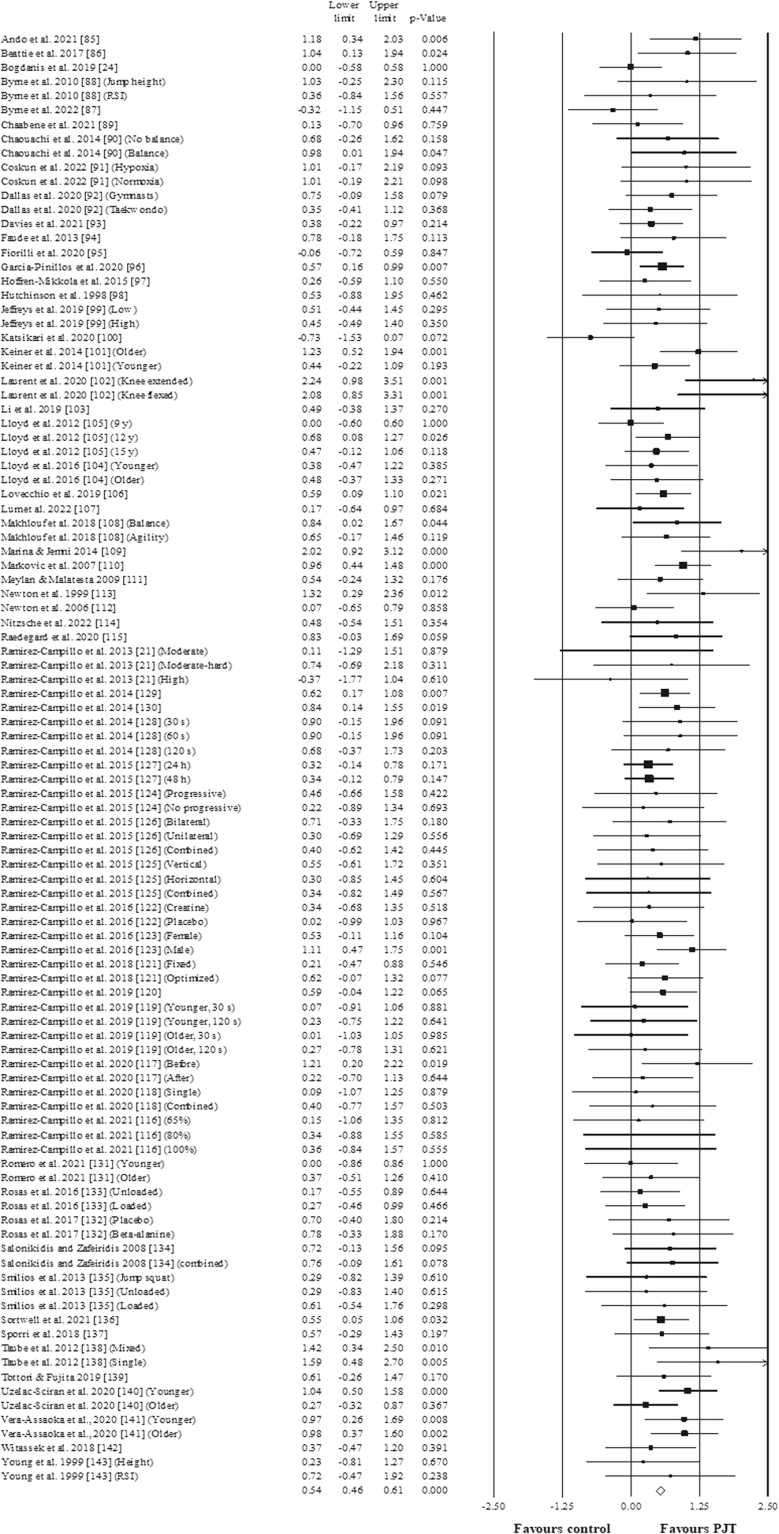
Forest plot illustrating plyometric jump training (PJT)-related improvements of the reactive strength index (RSI) in comparison to active/passive controls. Forest plot values are shown as effect sizes (ES [Hedges’ g]) with 95% confidence intervals (CI). Black squares: individual studies. The size represents the relative weight. White rhomboid: summary value. Mean results: ES (left column) = 0.54, 95% CI 0.46–0.62, *p* < 0.001, *I*^2^ = 0.0, *N* total participants = 2576, Egger test two-tailed = 0.365

Results (Fig. 2) showed a significant effect for the PJT groups compared to the active/specific-active control groups: ES = 0.54, 95% CI 0.46–0.62, *p* < 0.001, *I*^2^ = 0.0%, total participants *n* = 2576, Egger test two-tailed = 0.365. After the sensitivity analyses (automated leave-one-out analysis), the robustness of the summary estimates (i.e., *p* value, ES and 95% CI, *I*^2^) was confirmed.

#### Moderator Analyses

Regarding participants’ age, PJT-induced RSI changes were greater for adults (41 groups; ES = 0.67, 95% CI 0.53–0.81; *p* < 0.001; *I*^2^ = 1.8%) compared to youth (61 groups; ES = 0.47, 95% CI 0.37–0.57; *p* < 0.001; *I*^2^ = 0.0%), with a between-moderator category *p* value of 0.023.

Regarding the PJT programming variable total duration, greater RSI changes were noted after > 7 weeks (37 groups; ES = 0.66, 95% CI 0.50–0.83; *p* < 0.001; *I*^2^ = 32.0%) compared to ≤ 7 weeks (65 groups; ES = 0.47, 95% CI 0.37–0.57; *p* < 0.001; *I*^2^ = 0.0%), with a between-moderator category *p* value of 0.048.

With regard to the total number of PJT sessions, a trend was noted for greater RSI changes after > 14 PJT sessions (39 groups; ES = 0.65, 95% CI 0.49–0.82; *p* < 0.001; *I*^2^ = 29.1%) compared to ≤ 14 sessions (63 groups; ES = 0.47, 95% CI 0.37–0.57; *p* < 0.001; *I*^2^ = 0.0%), with a between-moderator category *p* value of 0.060.

In terms of PJT frequency, greater RSI changes were noted after using three weekly PJT sessions (18 groups; ES = 0.73, 95% CI 0.54–0.93; *p* < 0.001; *I*^2^ = 0.0%) compared to less than three sessions (84 groups; ES = 0.50, 95% CI 0.41–0.58; *p* < 0.001; *I*^2^ = 2.6%), with a between-moderator category *p* value of 0.027.

Regarding the total number of jumps completed during the PJT intervention, similar RSI changes were noted after ≤ 1080 (50 groups; ES = 0.51, 95% CI 0.39–0.63; *p* < 0.001; *I*^2^ = 0.0%) compared to > 1080 total jumps (51 groups; ES = 0.57, 95% CI 0.44–0.69; *p* < 0.001; *I*^2^ = 22.2%), with a between-moderator category *p* value of 0.536.

Concerning study randomization, similar RSI changes were noted for non-randomised studies (12 groups; ES = 0.80, 95% CI 0.42–1.18; *p* < 0.001; *I*^2^ = 58.1%) compared to randomised studies (85 groups; ES = 0.52, 95% CI 0.43–0.60; *p* < 0.001; *I*^2^ = 0.0%), with a between-moderator category *p* value of 0.153.

#### Meta-regression

The meta-regression analysis was computed for RSI including three training programming parameters (frequency, duration and total number of sessions). None of the training variables explained the effects of PJT on the RSI (*p* = 0.784, *p* = 0.714 and *p* = 0.984, respectively; *R*^2^ = 0.0).

#### Certainty of Evidence

Results of the GRADE analyses are provided in Table 4. Following previous recommendations [146], we chose seven outcomes for the analysis. According to the GRADE assessment, the certainty of evidence was considered moderate for the main analysis, and low-to-moderate across the moderator analyses.

**Table 4:** GRADE analyses

	No. of groups (*k*) and PSS	RoB in studies	Inconsistency	Indirectness	Imprecision	Risk of publication bias	Certainty of evidence
Main analysis							
PJT effects on RSI	*k* = 102, *n* = 2576	Low^a^	Moderate^b^	Low^c^	Low (small positive effect of PJT)^d^	Low^f^	Moderate
Moderator analyses							
Youths versus adults	*k* = 102, *n* = 2576	Low^a^	Moderate^b^	Low^c^	Low (greater benefits in adults)^d^	–	Moderate
> 7 versus ≤ 7 weeks program duration	*k* = 102, *n* = 2575	Low^a^	Moderate^b^	Low^c^	Low (greater benefits after > 7 weeks)^d^	–	Moderate
> 14 versus ≤ 14 training sessions	*k* = 102, *n* = 2576	Low^a^	Moderate^b^	Low^c^	Moderate (unclear direction of effects)^e^	–	Low
3 versus < 3 weekly sessions	*k* = 102, *n* = 2576	Low^a^	Moderate^b^	Low^c^	Moderate (unclear direction of effects)^e^	–	Low
≤ 1080 versus > 1080 jumps	*k* = 101, *n* = 2555	Low^a^	Moderate^b^	Low^c^	Moderate (unclear direction of effects)^e^	–	Low
Randomized versus non-randomised	*k* = 97, *n* = 2506	Low^a^	Moderate^b^	Low^c^	Moderate (unclear direction of effects)^e^	–	Low

*GRADE* Grading of Recommendations Assessment, Development and Evaluation, *PSS* pooled sample size, *RoB* risk of bias

^a^No downgrade of evidence as the median PEDro scores were at least high (≥ 6)

^b^Downgrade evidence by one level due to clinical heterogeneity (populations, interventions, comparators). No comparison presented high levels of statistical heterogeneity

^c^No downgrading. Eligibility criteria (not featured in the table) ensured appropriate populations, interventions, and outcomes (without the need to use proxies or surrogates)

^d^No downgrading, as ≥ 800 participants were available for a comparison and there was a clear direction of the effects

^e^Downgraded by one level, as ≥ 800 participants were available for a comparison but there was an unclear direction of the effects

^f^No downgrading (Egger’s test > 0.05)

#### Adverse Effects

Most of the included studies did not report soreness, pain, fatigue, injury, damage or adverse health effects related to the PJT intervention. One study indicated that one subject did not complete the intervention due to pain in the Achilles tendon, possibly due to PJT [99], and four studies indicated that subjects reported relatively reduced subjective muscle pain in their lower limbs after the initial training sessions (e.g. between 0 and 3, on a 10-point visual analogue scale), with a significant reduction during the last weeks of the PJT interventions [129–132].

## Discussion

The aim of this systematic review with meta-analysis was to examine the effects of PJT on the RSI of healthy individuals across the lifespan compared with active/specific-active controls. The meta-analysis indicated that PJT is effective at improving the lower-limb RSI in healthy individuals across the lifespan, with an overall ES = 0.54 (95% CI 0.46–0.62). Findings from this study are robust considering that the results are based on 61 articles with low risk of bias (good PEDro quality), low impact of study heterogeneity, moderate GRADE rating, and comprising 2576 participants. The main findings can be summarized as follows: PJT induced larger RSI improvements in adults versus youth, > 7 weeks of PJT were more effective than ≤ 7 weeks of training, three sessions per week resulted in larger effects compared with < 3 weekly sessions, > 14 total PJT sessions produced larger effects than ≤ 14 sessions.

### Moderators of Reactive Strength Index (RSI) and Plyometric Jump Training (PJT)

#### Participant Characteristics: Age

When the chronological age of the participants has been meta-analysed in relation to the physical fitness adaptations to PJT [33, 60], similar or even greater improvements have been noted among older participants. Indeed, the results of our meta-analysis indicated that RSI changes after PJT were greater for adults (ES = 0.67, 95% CI 0.53–0.81) compared with youth (ES = 0.47, 95% CI 0.37–0.57). Accordingly, other age-related factors appear more relevant to explain PJT-related RSI adaptations such as the biological maturity status of the younger participants. In youth, biological maturation has been under-researched as a potential moderator of the effects derived from PJT interventions. Amongst PJT studies that included youth, the maturity status was reported in only seven out of 34 (21%) studies. This research gap is common in the PJT literature [10] and resistance training studies in general [147]. Moreover, different maturation assessment techniques are used (e.g., pubic hair development, predicted age of peak height velocity), introducing heterogeneity across studies. Additionally, gold standard assessment techniques (e.g., skeletal age) [148–150] are rarely reported. Considering that physiological maturation may affect PJT-related RSI adaptations in both youth males and females [7, 78, 80], and considering that most of the studies included in this systematic review involved youth, future studies should attempt to overcome this methodological issue by examining youth participants. Alternatively, studies with youth populations may have used a more conservative PJT dosage, precluding RSI maximization. Currently, there is a lack of clear cut-off values for the prescription and progression of PJT programming parameters [151], or the use of adequate markers of PJT intensity [123, 152, 153], including the RSI [118, 154]. Future research should be conducted to solve these limitations which could help to maximize RSI in youth and adult populations, and to reduce potential adverse health events related to PJT programs. Of note, some of the included adult studies in our moderator analysis [114, 115] reported age as mean ± SD values (e.g., 19 ± 2 years). Closer scrutiny of the adult population revealed that primarily college students were recruited in these studies. Accordingly, it is possible that few (if any) participants were aged < 18 years. Overall, the moderator analysis comprised 41 adult and 61 youth groups. Therefore, and for the above-mentioned reasons, the number of studies that may have included participants aged < 18 years in the adult group was negligible.

#### PJT Programming Parameter: Total Duration

Regarding the PJT programming parameter total duration, greater PJT-related RSI changes were noted after > 7 weeks (ES = 0.66, 95% CI 0.50–0.83) compared to ≤ 7 weeks (ES = 0.47, 95% CI 0.37–0.57). In line with longer durations, a trend was noted for greater RSI changes after > 14 PJT sessions (ES = 0.65, 95% CI 0.49–0.82) compared to ≤ 14 sessions (ES = 0.47, 95% CI 0.37–0.57). These results are aligned with those from a meta-analysis regarding the effects of PJT on jump height in female soccer players, which demonstrated greater improvements after ≥ 8 weeks (ES = 1.24) compared to < 8 weeks (ES = 0.66) [59]. Similarly, among male youth soccer players, better 10-m linear sprint performances were noted after programmes > 7 weeks (ES = 0.93) compared to ≤ 7 weeks (ES = 0.11). Moreover, in PJT interventions that incorporated mid-study measurements, although improvements in physical fitness (i.e., linear sprinting, jumping, maximal strength) were noted after 4 weeks of PJT, larger improvements were observed after periods of 6, 8, 12 and 16 weeks of training [155, 156]. Collectively, although the evidence suggests that PJT may induce early adaptations in some outcomes of physical fitness, including the RSI, greater improvements are likely after longer-term interventions. However, although the duration of the training programs in the intervention groups ranged from 4 to 96 weeks, most studies lasted 6 weeks, with a median value of 7 weeks (i.e., cut-off value used for moderator analysis). Thus, there is a need for long-term PJT intervention studies in future research.

#### PJT Programming Parameter: Frequency

Regarding PJT frequency, greater PJT-related RSI changes were noted after three weekly PJT sessions (ES = 0.73, 95% CI 0.54–0.93) compared to < three sessions (ES = 0.50, 95% CI 0.41–0.58). There are several theoretical advantages of increased training frequency. For example, increased protein synthesis in response to training may last for 24–48 h in untrained individuals [157] and 24 h in trained individuals [158]. Consequently, a higher training frequency may provide more time for a net positive protein balance, thus enhancing muscular adaptations [159]. Similarly, greater weekly training frequency may favour bone mass accretion [160]. Furthermore, increased frequency of neuromuscular stimuli during a weekly training schedule may also help to optimise motor learning [161]. In addition, distributing the same weekly load across higher frequencies (i.e., several days) may reduce fatigue during the training sessions [159] and recovery duration between sessions [162]. Nonetheless, studies included in this meta-analysis that applied different PJT frequencies also applied a different total number of jumps. For example, one study applied three weekly PJT sessions over a period of 8 weeks [87] with an RSI improvement of ~ 54% after a total of 2400 jumps. In contrast, another study [90] applied two weekly PJT sessions over a period of 8 weeks, with a RSI improvement of ~ 20% after a total of 660 jumps. Contrary to our findings, previous results suggest that training frequency is a less decisive moderator when the training load is equated [163–165]. Indeed, a recent review [154] reported no effects of PJT frequency on soccer athletes’ athletic performance (e.g., jump height) when the weekly training load was equated. Moreover, two meta-analyses [59, 60] revealed no effects of PJT frequency on female and young male soccer players’ physical fitness (e.g., linear sprint, vertical jump). Furthermore, when the total number of jumps was equated, one or two weekly PJT sessions induced similar physical fitness improvements (e.g., linear sprint, jumping), irrespective of the participants’ age or sex [166–168]. Overall, it seems that when the weekly number of jumps is equated, training frequency seems not to affect training induced adaptations. However, when a greater number of jumps needs to be accumulated, a greater training frequency may allow some logistical advantages (e.g., greater inter-repetition rest, and training intensity) that could augment the training responses. In such cases, and considering the difficulty many coaches face to schedule more weekly training sessions, a pragmatic approach to increase PJT weekly frequency (and/or volume) may involve the integration of PJT exercises at the end of the warm-up of training sessions (e.g., composite training) [89], with the advantage of potentially increasing linear and change-of-direction speed movements [169–171]. Of note, our moderator analyses included studies that applied < three weekly PJT sessions compared to studies that applied three weekly sessions. Therefore, the maximum number of weekly sessions amounted to three in the included studies. If experimental studies [166–168] compared the PJT effects on participants’ physical fitness, by using different number of training sessions per week, the authors scheduled either one or two weekly sessions. A focus of future research may consider more than three weekly sessions.

#### PJT Programming Parameter: Total Number of Jumps

Regarding the total number of jumps completed during the PJT intervention, similar PJT-related RSI changes were noted after ≤ 1080 (ES = 0.51, 95% CI 0.39–0.63) compared to > 1080 total jumps (ES = 0.57, 95% CI 0.44–0.69). Of note, the applied total number of jumps across PJT programs varied widely among studies, in part due to the different duration of studies (i.e., 4 weeks vs. 96 weeks) or the type of jump exercises used (e.g., drop jump vs. jump rope), ranging from 108 up to ~ 21,000 total jumps. However, the optimal values are still yet to be determined, with some interventions prescribing training volumes in different ways such as duration, distance, repetitions (i.e., foot contacts, foot contacts per leg), or a mixture of these volume-indexes. To date, very few studies have included PJT groups with different volumes being prescribed to each group [126, 172–176]. From the aforementioned studies, only four [126, 172, 174, 175] provided an adequate comparison between groups using a different total number of jumps, and only one [126] observed greater physical fitness improvements after a greater total number of jumps. The reasons for the different findings are not clear at present though it is interesting to note the results of a recent meta-analysis that demonstrated that measures of stiffness (e.g., leg, joint, closely related to RSI [2, 177]) adaptations to PJT were greater when the applied dose was lower [178]. Overall, from the best available evidence (e.g., randomised, controlled studies), compared to greater total number of jumps (i.e., > 1080), a conservative total number of jumps (i.e., ≤ 1080) seems equally effective in improving RSI, over a period ≥ 4 weeks. Independent of this, some type of volume-based overload (e.g., number of training sessions per week, training exercises, training sets, training repetitions per set) may be needed to maximize improvements, with a relatively lower number of jumps at the beginning of the program and a progressive increase in number towards the end. Of note, progressive overload would need to consider PJT exercise intensity as well. For example, a high-volume to low-volume approach might be used when the intensity of PJT is increasing. In some instances, a tapering period may further maximize improvements [179]. From an injury prevention perspective, the current evidence points toward the use of a conservative number of jumps, which not only may allow significant RSI improvements, but also a lower risk of injury [180–182].

### Adverse Health Effects

One study indicated that one older adult (from total *n* = 20) did not complete the intervention due to pain in the Achilles tendon, possibly due to PJT [99], and four studies indicated that youth (mostly male soccer players) reported low levels of muscle pain in their lower limbs after initial training sessions [129–132]. However, most of the included studies did not report any adverse health events related to the PJT intervention. The relative safety of PJT programs has been previously supported [10, 17, 18]. Moreover, when adequately programmed and supervised, PJT interventions may also reduce the risk of injury [183, 184]. Although PJT seems to be safe, caution is recommended when applying this type of training in poorly conditioned participants with low strength levels or an inability to decelerate their body mass during landing tasks. Suggestions for progression during PJT have previously been provided by Lloyd et al. [185], Sáez de Villarreal and Ramirez-Campillo [186], and Flanagan and Comyns [5]. These recommendations can be used to improve physical fitness (including RSI) and mitigate the risk of sustaining injuries. For example, a line of progression may entail for vertical jumps: (1) drop lands, (2) drop jumps, (3) repeated hurdle jumps (low), (4) repeated hurdle jumps (high). For horizontal jumps a line of progression may contain the following exercises: (1) single leg hops, (2) repeated single leg hops, (3) straight leg bounding, (4) bounding.

Moreover, a higher number of repetitions of PJT exercises may be associated with increased injury risk, particularly in females [180, 182]. Of note, the moderator analysis computed in this systematic review revealed that a total number of jumps > 1080 or ≤ 1080 seems equally effective in improving the RSI. In addition, the periodic application of taper strategies (i.e., reduction in PJT volume) during a program can reduce overload-induced inflammation from large eccentric loads [187, 188]. Accordingly, a tapering strategy may help to avoid injuries and facilitate adaptive processes in the musculoskeletal system, optimising the RSI [179, 189]. Moreover, although none of the included studies reported adverse health events, none of the studies reported on participants’ movement quality during plyometric jump drills and progressive overload. Although the potential relationship between movement competency and PJT progression [185, 190, 191], and some potential factors associated with the safety of PJT drills [192–194], have been reported, conclusive evidence is lacking. Moreover, there is a lack of clear cut-off values for the prescription and progression of PJT [151], or the use of adequate markers of PJT intensity [123, 152, 153], including the RSI [118, 154]. To improve the RSI and to reduce any potential adverse events derived from PJT programs, the aforementioned issues should be further investigated.

### Limitations

First, regarding the risk of bias (methodological quality) of the included studies, according to the PEDro checklist, the median (i.e., non-parametric) score was 6.0 (low risk of bias—good quality). Nonetheless, although most of the included studies (*n* = 34) in our meta-analysis attained a low risk of bias, 27 studies did not score more than 5 points in the PEDro scale, with only two studies attaining ≥ 8 points. Previous systematic reviews that focused on PJT [56, 195, 196] and used the PEDro scale also suggested that the published PJT studies need to reduce the risk of bias. This finding is likely due to the difficulties in conducting studies related to the blinding of participants or therapists. Indeed, most of the included studies (*n* = 45–59) did not comply with PEDro items 3, 5, 6, and 7 (i.e., allocation concealment, blinding of participants, blinding of coaches, and blinding of assessors, respectively). Second, regarding potential adverse events derived from PJT interventions, even though the included studies did not specify any negative responses associated with the PJT intervention, it is unclear if there was an attempt by the researchers to comprehensively record all possible adverse events. Therefore, future studies are encouraged to be fully transparent regarding any injuries, pain or other adverse PJT-related events, and the methods used to assess these, including a register of the protocol. This would help to expand our knowledge on the safety of this type of training. Third, regarding participants' sex, 17 groups involved females only (*n* = 385, 15% of total participants). The lower number of females compared to males is unfortunately relatively common in the PJT literature [10, 17]. The reason why females are less involved in PJT research is probably multifactorial and not only related to PJT but overall to strength and conditioning research [147, 197–199]. Likely reasons could be that for many years fewer females have practiced professional sports (e.g., soccer, handball, track and field) that benefit from PJT compared to males. On a global level, cultural and/or religious reasons may have reinforced this phenomenon. In addition, PJT and power exercises in general may not have been within the scope of coaches dealing with exercising females. The positive effects of PJT exercises for females could be less recognised by coaches, and researchers have neglected this topic for many years and increased their research efforts only recently. There is evidence [200] that it takes up to 17 years until research findings are translated into (clinical) practice. Such a limitation is applicable to studies in athletes as well, such as in female soccer players. Indeed, in the current systematic review most (*n* = 22) of the studies that recruited athletes included soccer players, although only three out of 22 soccer studies included females. With the increased participation of females in sports (e.g., 50% increase in the number of female soccer players was observed between 2000 and 2006 [201]), research is required to enhance knowledge with regards to PJT programming for RSI optimization in female athletes. Relatedly, it seems that the number of female athletes involved in sports as well as the number of studies conducted in the general female population and female athletes is increasing steadily [197]. Fourth, some sports already include a considerable jumping load in their sport-specific actions (e.g., long jump, high jump, basketball). Accordingly, when programming PJT in these sports, the additional sport-specific demands in terms of jump load must be considered. However, due to methodological reasons, we were unable to conduct a meta-analysis on the effects of PJT on the RSI according to the type of sport. The main methodological reason that precluded a sport-specific meta-analysis was the considerable difference in the number of studies that provided data for specific sports: soccer *n* = 22, endurance runners *n* = 5, gymnasts *n* = 4, volleyball *n* = 2, handball *n* = 1, hurling *n* = 1, tennis *n* = 1, and rugby *n* = 1. Considering general [83–86] and PJT-specific [61] recommendations, the certainty of evidence would be considered very low for outcomes or moderators not included in meta-analyses. Therefore, current evidence for recommendations on the potential differences for the effectiveness of PJT on the RSI, according to the type of sport, would be rated as very low. Fifth, given the large difference in the number of studies that included active compared to specific-active controls, and the low (*n* < 10) number of studies that included specific-active controls, a moderator meta-analysis on the type of controls was precluded, due to a potentially biased comparison arising from analyses including < 10 studies per characteristic being modelled, particularly when the covariates are unevenly distributed across studies [202]. Indeed, participants involved in PJT attained a significant different RSI change when compared to active controls (66 groups [94 when proportionally divided for studies that included multi-PJT groups]; ES = 0.56, 95% CI 0.48–0.64; *p* < 0.001; *I*^2^ = 0.0%), although not when compared to specific-active controls (seven groups; ES = 0.29, 95% CI − 0.09 to 0.67; *p* = 0.139; *I*^2^ = 34.6%), with a between-moderator category *p* value of 0.171. Therefore, although PJT might be similarly effective compared to other training approaches to improve the RSI, more studies are required to assess the effectiveness of PJT in comparison with other training protocols. Of note, out of the 73 control groups, only seven groups were specific-active controls, which means that they were involved in a non-PJT intervention (e.g., resistance training).

### Practical Applications and Directions for Future Research

#### Sample Size

Small sample sizes represent an often encountered limitation in the sport science literature [203], including the PJT literature [10, 17], particularly when examining athletes. Although smaller studies may implement more thorough interventions than larger trials [73, 204–206], they exhibit larger effects (type I error, i.e., false positives) [73, 204, 206–208]. In PJT studies ~ 10 participants are usually included per study group [10, 17], casting doubts on the transferability of PJT findings into practice. Indeed, from the 61 studies included in our meta-analysis, a mode of ten participants per PJT group was observed, with a median number of 11 participants, and a mean of 14.8 participants per PJT group. Future studies should conduct a priori power analysis to estimate the required sample size and to increase the robustness of the statistical power [203]. Free online software tools and guidelines are available to compute a priori power analyses, including specific recommendations for sport sciences [203, 209]. Small sample sizes are often encountered in sport science, particularly when working with elite athletes. The computation of interindividual variability may offer great value when dealing with small study samples [210–213]. A few studies included in our systematic review [106, 113, 131] provided inter-individual analyses for the adaptive response of the RSI to PJT interventions. Researchers conducting studies in elite sports with small samples are advised to calculate reliability or typical error data on an individual level (i.e., individual target scores) [2], in comparison to the use of arbitrary smallest worthwhile change values. For example, if the RSI from an individual is equal to 2.8 and its coefficient of variation for RSI is 6%, therefore: 2.8 × 0.06 (6% as a decimal) = 0.168. Then, 2.8 + 0.168 = 2.968. As such, an athlete, for example, needs to have scored > 2.968 as an RSI improvement to achieve a true improvement, which is greater than the noise of the test.

Of note, while in some fields the outcomes are sensitive to randomization, in others it may not be necessary. Indeed, for randomised (ES = 0.52) versus non-randomised (ES = 0.80) studies in this field, we noted a lack of identifiable differences in direction of the outcomes. Therefore, in the case of assessing PJT effects on RSI, randomization may not be a key factor. Nonetheless, researchers should aim to conduct future studies to address the effects of PJT on RSI using adequate sample-size randomised, controlled trials.

#### Implications for Measurement and Assessment

Regarding the measurement and assessment of RSI, irrespective of the jump task, participants are usually required to perform a maximal jump displacement (either vertical or horizontal) and to minimize GCT [2, 19]. Although several measures of RSI are possible (e.g., modified RSI) with advanced laboratory equipment (e.g., force platforms, electromyography), for practical purposes in most field-based studies, the RSI is calculated via the division of jump height or flight time by the respective GCT, which can be assessed with low cost and versatile equipment, such as jump mats or mobile phones apps [2, 3, 214]. Indeed, most of the included studies in the meta-analysis used testing protocols involving drop jumps (47 studies) performed mostly (42 studies) on contact mats (including optical-based mats). Relatedly, most protocols (58 of 61 studies) were assessed using plyometric jump-specific tests (e.g., drop jump, vertical hop). Future PJT studies may assess RSI with other tests (e.g., speed, change of direction) to determine how well PJT transfers to RSI in other skills or capacities [122, 215–219].

Furthermore, the type of RSI reported in the studies included were mm/ms (*n* = 25), m/s (*n* = 9), cm/s (*n* = 8), cm/ms (*n* = 4), ms/ms (*n* = 3), s/s (*n* = 1), and unreported (*n* = 11). Considering that the RSI is a ratio, future researchers are encouraged to report RSI unit-less, as opposed to common reporting formats such as mm/ms (i.e., velocity). Additionally, for future studies, authors are encouraged to report not only the RSI, but its constitutive components as well (e.g., jump height, GCT). This would help to determine the magnitude of RSI improvement due to changes in one or more of its components. Relatedly, future researchers may consider the measurement of the RSI constitutive components jump height and GCT, but also countermovement depth, for a more comprehensive view of potential adaptations. Although RSI measurement is usually reliable [2], the countermovement performed during jumps may be an important confounding factor for RSI determination, stressing the need for adequate technique mastering and familiarisation with the test procedures before RSI measurements [26].

## Conclusions

Interventions involving PJT are more effective for improving RSI in healthy individuals across the lifespan compared to active/specific-active control conditions involving traditional sport-specific training as well as alternative training interventions. This conclusion is derived from 61 articles with low risk of bias (good methodological quality), low study heterogeneity, and a moderate certainty of evidence according to GRADE rating, comprising 2,576 participants. The observed PJT-related RSI changes were greater for adults compared with youth. Larger effects were found after > 7 weeks compared with ≤ 7 weeks of training. Three weekly exercise sessions were more effective than < three sessions, and > 14 total PJT sessions showed larger effects than ≤ 14 sessions.

## Supplementary Information

Below is the link to the electronic supplementary material.

Supplementary file1 (DOCX 33 kb)

## Declarations

### Availability of data and material

All data generated or analysed during this study are included in the article as Table(s), Figure(s), and/or Electronic Supplementary Material(s). Any other data requirement can be directed to the corresponding author upon reasonable request.
