# Supplementary material for: Effects of Plyometric Jump Training on the Reactive Strength Index in Healthy Individuals Across the Lifespan: A Systematic Review with Meta-analysis
Source: Sports Med. 2023 Mar 11;53(5):1029–53. doi: 10.1007/s40279-023-01825-0 (PMC10115703; doi:10.1007/s40279-023-01825-0)
Supplement: Supplementary file 1 — Supplementary file1 (DOCX 33 kb) [file 40279_2023_1825_MOESM1_ESM.docx]

**Electronic Supplementary Material Table S1**

**Article title**: Effects of plyometric jump training on the reactive strength index in healthy individuals across the lifespan: a systematic review with meta-analysis

**Author names**: Rodrigo Ramirez-Campillo, Rohit K. Thapa, José Afonso, Alejandro Perez-Castilla, Chris Bishop, Paul J. Byrne, Urs Granacher

**Affiliation and e-mail of the corresponding author**:

Prof. Urs Granacher, PhD

University of Freiburg, Department of Sport and Sport Science, Exercise and Human Movement Science, Sandfangweg 4, 79102 Freiburg i. Br., Germany. Email: [urs.granacher@sport.uni-freiburg.de](mailto:urs.granacher@sport.uni-freiburg.de); ORCID: 0000-0002-7095-813X

Table S1. Search strategy (code line) for each database and background of search history.

| **Date of the search** | April, 2017 | May, 2019 | August, 2021 ^c^ |
| --- | --- | --- | --- |
| **Databases** | PubMed | PubMed, WOS (Core Collection), Scopus | PubMed, WOS (Core Collection) ^a^, Scopus |
| **Keywords** | “plyometric”, “training” | “ballistic”, “complex”, “cycle”, “explosive”, “force”, “plyometric”, “shortening”, “stretch”, “training”, “velocity” | “ballistic”, “complex”, “cycle”, “explosive”, “force”, “jump”, “plyometric”, “power”, “shortening”, “stretch”, “training”, “velocity” |
| **Database fields for the search** | All | PubMed: all  WOS: all  Scopus: title, abstract, keywords | PubMed: all ^b^  WOS: all ^b^  Scopus: title, abstract, keywords ^b^ |
| **Restrictions for the search** | None | None | None |
| **Examples of search strategy code line** | PubMed: "plyometric exercise"[MeSH Terms] OR ("plyometric"[All Fields] AND "exercise"[All Fields]) OR "plyometric exercise"[All Fields] OR ("plyometric"[All Fields] AND "training"[All Fields]) OR "plyometric training"[All Fields]  WOS: (ALL=(plyometric)) AND ALL=(training)  Scopus: TITLE-ABS-KEY ( plyometric AND training ) | | |
| ^a^: except for the keywords “jump” and “power” searched in all WOS databases; ^b^: except for the keywords “jump” and “power” searched in the database field TITLE (a very poor efficiency was obtained in the search for results with the incorporation of other database fields); Note: after formal database search, the list of included articles and the inclusion criteria (see Table 1) were sent to three independent world experts in the field of plyometric-jump training (https://www.expertscape.com/ex/plyometric+exercise) to help identify additional relevant articles. The experts were not provided with our search strategy, to avoid biasing their own searches. Upon completion of all these steps, the databases were again consulted in search for errata or retractions of any included study; ^c^: After an initial search in April 2017, an account was created by one of the authors in each of the respective databases, through which the author received automatically generated email alerts regarding the search terms used. The search was refined in May 2019 and August 2021, with updates received daily (if available). Studies were eligible for inclusion, from inception in each database, up to May 2022. | | | |

**Electronic Supplementary Material Table S2**

**Article title**: Effects of plyometric jump training on the reactive strength index in healthy individuals across the lifespan: a systematic review with meta-analysis

**Author names**: Rodrigo Ramirez-Campillo, Rohit K. Thapa, José Afonso, Alejandro Perez-Castilla, Chris Bishop, Paul J. Byrne, Urs Granacher

**Affiliation and e-mail of the corresponding author**:

Prof. Urs Granacher, PhD

University of Freiburg

Department of Sport and Sport Science

Exercise and Human Movement Science

Sandfangweg 4

79102 Freiburg i. Br.

Germany

Email: urs.granacher@sport.uni-freiburg.de

ORCID: 0000-0002-7095-813X

Table S2. Additional exclusion criteria.

| Excluded were books, book chapters, and congress abstracts, as well as cross-sectional and review papers, and training-related studies that did not focus on the effects of plyometric-jump training (PJT) exercises, such as plyometric training without the use jumps (e.g., upper-body plyometrics only). Also excluded were retrospective studies, prospective studies (e.g., relationship between bone density at the end of PJT, and at several years of follow-up), studies in which the use of PJT exercises was not clearly described (e.g., authors stated “plyometric exercises were implemented”, without further explanation), studies for which only the abstract was available, case reports, special communications, letters to the editor, invited commentaries, errata, studies with doubtful quality or unclear peer-review process from the journal, overtraining studies, and detraining studies. In the case of detraining studies, these were considered for inclusion if involved a training period prior to a detraining period. |
| --- |
